# Fabrication and Applications of Solid-State Nanopores

**DOI:** 10.3390/s19081886

**Published:** 2019-04-20

**Authors:** Qi Chen, Zewen Liu

**Affiliations:** Institute of Microelectronics, Tsinghua University, Beijing 100084, China; chenqi14@mails.tsinghua.edu.cn

**Keywords:** solid-state nanopores, fabrication and shrinking technologies, applications of solid-state nanopores

## Abstract

Nanopores fabricated from synthetic materials (solid-state nanopores), platforms for characterizing biological molecules, have been widely studied among researchers. Compared with biological nanopores, solid-state nanopores are mechanically robust and durable with a tunable pore size and geometry. Solid-state nanopores with sizes as small as 1.3 nm have been fabricated in various films using engraving techniques, such as focused ion beam (FIB) and focused electron beam (FEB) drilling methods. With the demand of massively parallel sensing, many scalable fabrication strategies have been proposed. In this review, typical fabrication technologies for solid-state nanopores reported to date are summarized, with the advantages and limitations of each technology discussed in detail. Advanced shrinking strategies to prepare nanopores with desired shapes and sizes down to sub-1 nm are concluded. Finally, applications of solid-state nanopores in DNA sequencing, single molecule detection, ion-selective transport, and nanopatterning are outlined.

## 1. Introduction

Nanopore analysis technology originated from the invention of the Coulter counter and the recording technique of a single channel current. A nanopore embedded in a film is immersed in an electrolyte solution, dividing the solution into two reservoirs. The electric potential applied between two reservoirs generates an electric field across the pore, driving charged ions or molecules to pass through the nanopore. Since the first research application of biological nanopores for bio-sensing in 1996 [1], nanopore-based sensors have been widely studied by researchers [2,3,4]. Biological nanopores, such as α-hemolysin, *Mycobacterium smegmatis* porin A (MspA), and DNA packaging motors (like bacillus phage phi29 and bacteriophage T7), have great potential in the sensing of various analytes, including DNA, RNA, proteins, and so forth [1,3,5,6,7,8]. Advantages of these biological nanopores are the reproducibility at the atom level, stability in a wide pH range (pH 2–12), ease of surface functionalization, and suitable pore sizes for molecule translocation [9]. Diameters in the narrowest regions of α-hemolysin and MspA are 1.4 nm and 1.2 nm, respectively. As a result, they have been utilized for translocation of ssDNA (~1 nm in diameter) [6,9]. DNA packaging motors, like phi29 and T7, have larger diameters in the narrowest regions (3.6 nm and 3.9 nm, respectively), thus they have the capability of sensing molecules with larger feature sizes [10,11,12]. However, biological nanopores have some inherent drawbacks, such as the fixed pore shape and size, poor mechanical stability, and sensitivity to experimental environmental conditions (i.e., pH, temperature) [3,13]. In the early stages of development, the ultimate drawback of biological nanopores was the difficulty to scale pores to a large (1 M) array size. Now, Genia Technologies is on the way to achieving this by presenting a nanopore-based sequencing-by-synthesis (Nanopore-SBS) approach [14,15]. Based on the complementary metal–oxide–semiconductor (CMOS) chip developed by Genia Technologies (Mountain View, CA, USA, the nanopore-coupled polymerase enables highly scalable, single-molecule DNA sequencing. Follow-up work led to the emergence of solid-state nanopores in the early 21st century [16,17,18,19,20]. Solid-state nanopores have advantages over their biological counterparts, such as the stronger thermal, mechanical, and chemical stability; ease of modifications; tunable pore size and morphology; readily able to be integrated into nanofluidic or other nanodevices; and, more importantly, the scalability of fabrication [21,22].

**Figure 1:**
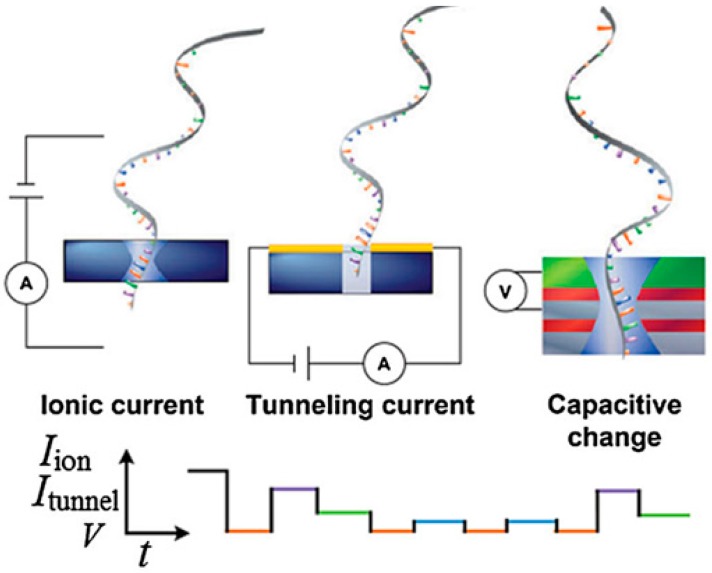
Principles of three detection methods of nanopore-based DNA sequencing (reprinted with permission from [22], copyright 2015, Science China Press). The upper panel presents schematics of measurements of the blocked ionic current (left), tunneling current (middle), and capacitive change (right). The lower panel is the time-varying signal.

Figure 1 demonstrates the principles of three detection methods of nanopore-based DNA sequencing. Blocked ionic current detection is the most mainstream and direct sequencing strategy based on both solid-state and biological nanopores [23,24,25]. A membrane embedded with a nanopore is sandwiched between two reservoirs, and a bias voltage is applied across the membrane. DNA or RNA molecules are added to the negatively biased reservoir filled with electrolyte solution, where they will electrophoretically pass through the nanopore. When the polymer chain crosses the nanopore, each type of nucleotide will generate a blocked ionic current with the unique magnitude of (ΔI) and a dwell time (Δt). As a result, de novo DNA sequencing can be theoretically realized. However, it is very difficult to discriminate among nucleotides in a moving DNA chain, since four types of nucleotides are different in the structure by only a few atoms [26]. Besides, the rapid DNA translocation speed and limited spatial resolution are challenges to distinguish each nucleotide. To improve the accuracy of recognition, the transverse tunneling current detection method based on solid-state nanopores was developed [27,28]. In this method, the tunneling current is only generated by the nucleotide inside the gap between a pair of electrodes. The chemical structure and lateral conductance of each type of nucleotide is unique, resulting in differences in the tunneling current [27,28,29,30,31]. However, the tunneling current is greatly affected by the direction of the nucleic acid molecule to be tested [3]. The capacitance variation detection method is an alternative method of DNA sequencing. The principle of capacitance variation detection is the use of nanopores of metal-oxide-semiconductor (MOS) structures [32,33]. When DNA passes through the nanopore driven by an external voltage, the capacitor is polarized. The upper and lower conductors serve as electrodes to record potential changes caused by each single nucleotide passing through the nanopore. Nucleotide discrimination is realized based on the unique electrostatic charge distribution of each type of nucleotide. High-speed operation is feasible during capacitance variation detection because of the high-speed performance of the embedded MOS field-effect transistor (MOSFET), but the noise induced by random variations in the temperature and molecular motion reduce the measurement accuracy [34]. Biological nanopores are not able to work via measurements of the tunneling current and capacitance variation, because the fabrication of electrodes is necessary.

Apart from DNA sequencing, solid-state nanopores have attracted growing attention in molecular separation [35,36], energy conversion [37], and ion logic circuits [38], to mention a few. As a fundamental issue, the fabrication of solid-state nanopores inspired extensive studies. Extensive reviews on the fabrication of nanopores have been published by various groups [39,40,41]. Although technologies, such as focused ion beam (FIB) and focused electron beam (FEB) drilling, are widely used, they can only fabricate nanopores one by one on thin membranes with thicknesses < 100 nm. Thus, a great number of techniques have been developed to fabricate solid-state nanopores with different minimum pore sizes, pore inner morphologies, and production scales. In this review, we discuss and compare the performances of the existing fabrication methods to achieve the goal of achieving cost-effective and scalable manufacturing uniform nanopore arrays. In addition, some typical applications of solid-state nanopores are outlined.

## 2. Fabrication Strategies of Solid-State Nanopores

There are many techniques to fabricate solid-state nanopores. For instance, FIB and FEB drilling methods, and dielectric breakdown methods have capabilities of fabricating <5 nm nanopores on suspended films. Chemical solution strategies are utilized for massive production at low-cost. Some other scalable fabrication technologies are proposed as well with diverse nanopore performances. Here, a classification of the fabrication strategies reported to date is given.

### 2.1. FIB and FEB Drilling Technology

FIB and FEB can be used for directly drilling small nanopores on suspended films. Using these techniques, nanopores with various shapes and sizes have been manufactured on a lot of materials, including SiN [16,42,43,44], SiO_2_ [45], SiC [46], metal [47], and 2D material [48] films.

**Figure 2:**
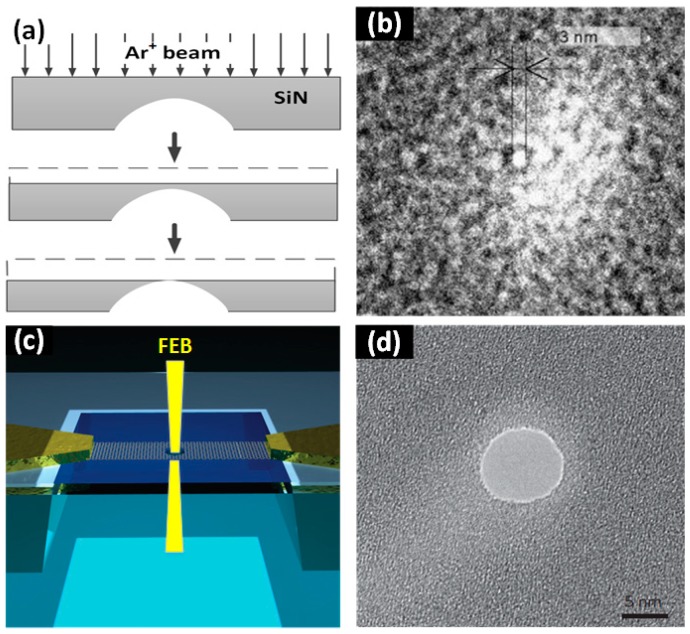
Focused ion beam (FIB) and focused electron beam (FEB) drilling methods. (**a**) Schematic of the FIB drilling technique (reprinted with permission from [52], copyright 2001, Springer Nature). (**b**) A 2.5 nm diameter SiC nanopore fabricated using FIB drilling (reprinted with permission from [46], copyright 2007, Elsevier B.V.). (**c**) Schematic of the FEB drilling technology. An FEB from the TEM drilled a nanopore directly on a suspended graphene membrane. (**d**) A TEM image of a graphene nanopore ((**c**,**d**) are reprinted with permission from [56], copyright 2013, Springer Nature).

FIB drilling is widely used to fabricate nanopores [49,50,51]. Figure 2a demonstrates the schematic of FIB drilling technology to fabricate an SiN nanopore [52]. A bowl-shaped cavity was created by reactive ion etching. Materials from the surface of the SiN membrane were removed under irradiation of a focused Ar^+^ beam, until the bottom of the bowl-shaped cavity was exposed, thus forming a nanopore. Gierak et al. [53] fabricated nanopores with diameters < 5 nm using 30 keV Ga^+^ ions. Further, they combined an improved direct writing system to fabricate a 2.5 nm nanopore in an SiC film [46], as shown in Figure 2b. A helium ion microscope (HIM) has a sub-nanometer probe, which can be used to fabricate nanopores with smaller diameters. With the help of an HIM, nanopores with minimum diameters of 1.3 nm were manufactured [54].

FEB drilling is also a popular technology to directly drill nanopores on a suspended membrane based on the atomic-scale erosion of the membrane under FEB, as shown in Figure 2c. Shim et al. [55] used FEB to drill 2 nm nanopores on HfO_2_ films. Wu et al. [47] used FEB from a transmission electron microscope (TEM) to drill polygonal nanopores (3 to 8 nm in diameter) with different shapes on magnesium (Mg) film. In 2008, Michael et al. [49] used FEB drilling technology to prepare nanopores with diameters of ~3.5 nm on a graphene membrane. After that, FEB drilling has become the first choice for the manufacturing of nanopores with ultra-small dimensions on 2D membranes, such as graphene [56], boron nitride (BN) [57], and MoS_2_ [58]. 

FIB and FEB can directly drill <5 nm nanopores. However, nanopores are drilled one by one, and the manufacturing process requires professional operations. Moreover, they are not cost-effective due to the requirement of expensive instruments, such as a FIB and TEM.

### 2.2. Dielectric Breakdown Technology

**Figure 3:**
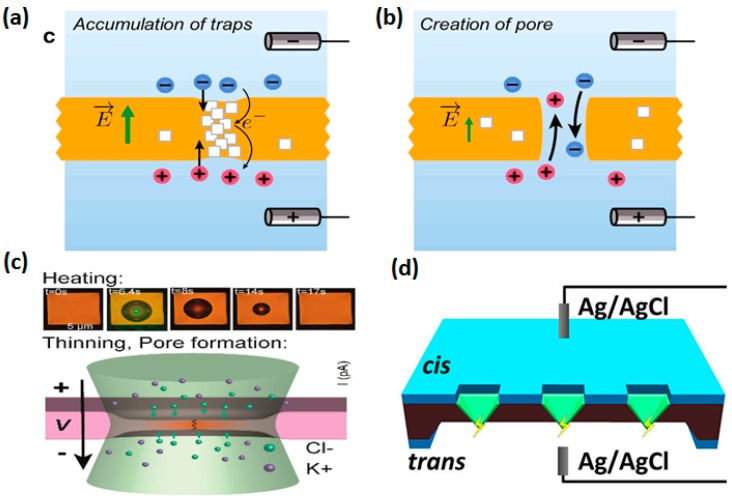
Schematic of the dielectric breakdown method. (**a**,**b**) show nanopore formation based on the accumulation of random traps (reprinted with permission from [59]). (**c**) Photothermally assisted thinning of the SiN membrane to prepare nanopores on a localized heating region (reprinted with permission from [62], copyright 2018, American Chemical Society). (**d**) Schematic of pore formation at tips of inverted-pyramidal membranes using the high voltage induced dielectric breakdown method (reprinted with permission from [63], copyright 2018, American Chemical Society).

To reduce the manufacturing cost and simplify the fabrication process, dielectric breakdown technology has been proposed. By applying a high breakdown electric field across the membrane, traps inside the membrane tend to generate and accumulate over time, thereby forming nanopores [59,60,61] (Figure 3a,b). In 2014, Kwok et al. [59] firstly reported research on the fabrication of solid-state nanopores with diameters of ~2 nm using the dielectric breakdown method. Ag/AgCl electrodes were placed in both reservoirs to apply a constant high electric field and record the current across the membrane. Nanopore generation was confirmed by a sudden rise in the current to a pre-determined cut-off current. However, a large leakage current across the film was generated under the high electric field, which affected the confirmation of the nanopore formation. Kwok et al. [59] measured a leakage current of 30 to 80 nA on a 10-nm-thick SiN membrane, when the cut-off current was 130 nA and the bias voltage was 5 V. Additionally, when the bias voltage increased to 15 V, the leakage current on a 30-nm-thick SiN membrane was ~50 nA when the cut-off current was 90 nA. To weaken the influence of the leakage current, a technique called multilevel pulse-voltage injection (MPVI) was proposed by Yanagi et al. [60] to fabricate nanopores with sizes of sub-1 to 3 nm. MPVI is an iteration sequence composed of applied high and low voltage pulses. High voltage pulses (7 V) are used for nanopore generation, and low voltage pulses (0.1 V) are used to measure the current [60]. However, traps are randomly distributed in the membrane, which makes the amount and location of the pore uncontrollable.

To solve the positioning problem, multiple strategies have been proposed by many groups. As shown in Figure 3c, photothermally assisted thinning of the SiN membrane was used by Yamazaki et al. to obtain single nanopores on specified locations [62]. A visible wavelength laser beam was applied to generate efficient localized heating on a SiN membrane, resulting in the possibility of trap accumulation in the heated region. Recently, a self-aligned fabrication method was put forward by our group [63,64]. As shown in Figure 3d, pyramidal HfO_2_ and SiO_2_ membranes were fabricated by atomic layer deposition (ALD) and thermal oxidation inside inverted-pyramidal Si structures, followed by back-side thinning of Si to expose the tips of the pyramidal membranes. The maximum electric field was generated in the tips of the pyramidal membranes. As a result, nanopores formed in and near the tips. By using this method, nanopores with feature sizes ranging from 5 nm to 50 nm were successfully manufactured. 

The dielectric breakdown method is a simple and convenient fabrication technique to prepare nanopores without the requirement of costly instruments. Nanopore formation and enlargement could be easily achieved by applying a breakdown voltage across the membrane. Nevertheless, it can only fabricate single or multiple nanopores at a time, which lacks the capability of scalable production.

### 2.3. Chemical Solution Etching Technologies

As discussed before, FIB and FEB drilling methods have successfully fabricated <5 nm nanopores. However, they are not cost-effective, because they rely on serial manufacturing steps as well as on expensive instruments, such as FIB and TEM. In contrast, chemical solution etching enables low-cost and high-efficient solid-state nanopore preparation on a massive scale. Until now, chemical solution etching strategies used in solid-state nanopore fabrication include feedback-controlled wet etching [65,66], ion-track etching [67,68,69], electrochemical anodization [70,71,72,73], and metal-assisted chemical etching (MaCE) [74,75,76,77].

#### 2.3.1. Feedback-Controlled Wet Etching

**Figure 4:**
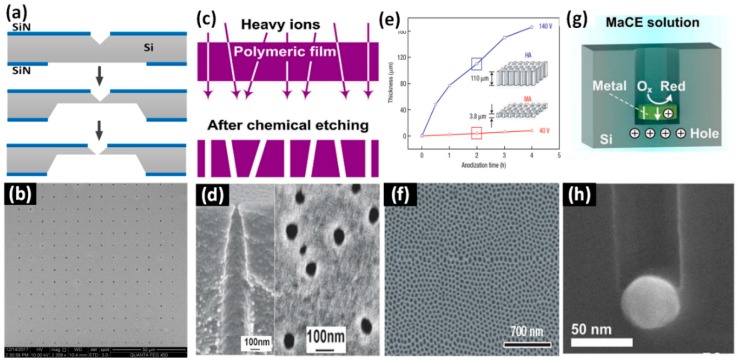
Chemical solution etching-based fabrication methods. (**a**) Schematic of the nanopore fabrication process, and (**b**) a scanning electron microscope (SEM) image of a nanopore array prepared by using the wet etching method (reprinted with permission from [66], copyright 2018, IOP Publishing). (**c**) Schematic of the track etching method. (**d**) SEM images of PET membranes with profiled pores (left, cross-sectional morphology of a single pore; right, surface of a membrane with a pore density of 3 × 10^9^ cm^−2^) (reprinted with permission from [82], copyright 2007, IOP Publishing). (**e**) Film thickness vs. time during anodization of electropolished aluminum substrates in 0.3 M H_2_C_2_O_4_ (1°C), and (**f**) an SEM image of Al_2_O_3_ nanopore arrays formed by mild anodization (MA) for 2 hours (reprinted with permission from [71], copyright 2006, Springer Nature). (**g**) Schematic of the Metal-Assisted Chemical Etching (MaCE) technique, and (**h**) an SEM image of an Si nanopore with a diameter of ~50 nm fabricated by MaCE (reprinted with permission from [84], copyright 2007, Elsevier Ltd.).

Wet etching in KOH solution combined with ionic current feedback [65] or color feedback [66,78] is a simple way to fabricate ordered nanopore arrays at the wafer scale. Figure 4a illustrates a schematic of the wet etching method used to manufacture nanopores by our group [66]. A 14 × 14 nanopore array with an average size of 30 nm was successfully obtained, as shown in Figure 4b. Nanopores obtained by this method exhibit a truncated-pyramidal shape with an inclined angle of 54.74°. However, it is difficult to achieve size uniformity because of the thickness variation in the Si wafer, bubbles in the KOH solution, and the limited precision of the standard photolithography [66]. The use of SOI wafers is an optional way to improve the pore size uniformity in an array, since the buried oxide offers a smooth surface. Wet etching enables fast and cheap fabrication of nanopores on a large scale, but the available nanopore sizes are relatively large, and the size uniformity needs to be improved.

#### 2.3.2. Ion-Track Etching

Ion-track etching is another simple way to fabricate nanopore arrays on polymeric substrates. The principle of the ion-track etching method is to create tracks by shooting high-energy heavy metal ions into a polymeric membrane, and then etch the materials where tracks are formed (Figure 4c). In chemical solutions, the etch rates near tracks are faster than the undamaged regions. By using the ion-track etching method, nanopores have been successfully fabricated in many polymeric films, such as polyimide [67], polycarbonate [68], and ethylene terephthalate [69]. These polymeric films have superior electrical insulation properties over semiconductors and metals. Besides, the pore morphology is typically conical-shaped with a high aspect ratio, because the polymeric film is usually several micrometers in thickness. However, the thicknesses of SiN, SiO_2_, and graphene membranes for nanopore fabrication by FIB and FEB drilling methods range from the sub-nanometer to tens of hundreds of nanometers. Asymmetric structures with high aspect ratios have been utilized to mimic protein or biological channels, and study ionic current rectification (ICR) properties [79,80,81]. Apel et al. [82] fabricated conical nanopore arrays with an average size of 51 nm and a pore density of 3 × 10^9^ cm^−2^, as shown in Figure 4d. Individual nanopores with minimum diameters of 2 nm were achieved using the ion-track etching method [83]. However, this method can fabricate nanopores only in polymeric films. The prepared nanopore arrays exhibit uneven distribution due to the uncontrollable generation of ion tracks. Moreover, it needs costly heavy ion accelerometers.

#### 2.3.3. Electrochemical Anodization

Electrochemical anodization enables the fabrication of nanopore arrays with a higher density. It originated from the discovery of self-assembled nanoporous alumina films by Masuda et al. in 1995 [72]. Now, it has become a common method to prepare high-density nanopore arrays on a variety of metal oxide materials [70,71]. Titanium dioxide (TiO_2_) nanopores with minimum diameters of 23 nm and high densities (5 × 10^10^ cm^−2^) have been successfully manufactured [73], as shown in Figure 4e–f. The size and location distribution of the nanopores are related to the applied voltage, etchant concentration, and substrate material. It is also hard to fabricate uniform nanopore arrays with diameters less than 10 nm.

#### 2.3.4. Metal-Assisted Chemical Etching (MaCE)

The MaCE technique was first proposed in 1997 to fabricate Si nanopores [77]. The Si covered with metal particles is etched faster than uncovered Si in an etchant solution (Figure 4g). As a result, the metal particles sink into the Si substrate, generating nanopores. MaCE has successfully been applied to fabricate nanopores with different geometries, such as cylindrical, conical, and helical structures [74,75,76]. The MaCE method can fabricate conical nanopores with very high aspect ratios. With the help of Ag catalysts, nanopores with diameters of 50 nm were obtained [84], as shown in Figure 4h. The MaCE technique is limited to the fabrication of nanopores in Si substrates. The generation of hydrogen during the etching process influences the etch rate of Si, resulting in the non-uniformity of the pore size.

In general, as one of the most commonly used methods for micro- and nanofabrication, chemical etching strategies allow massive production of solid-state nanopores with low cost. However, it is difficult to prepare ordered nanopore arrays with nanometer precision.

### 2.4. EBL-Assisted Etching Technologies

**Figure 5:**
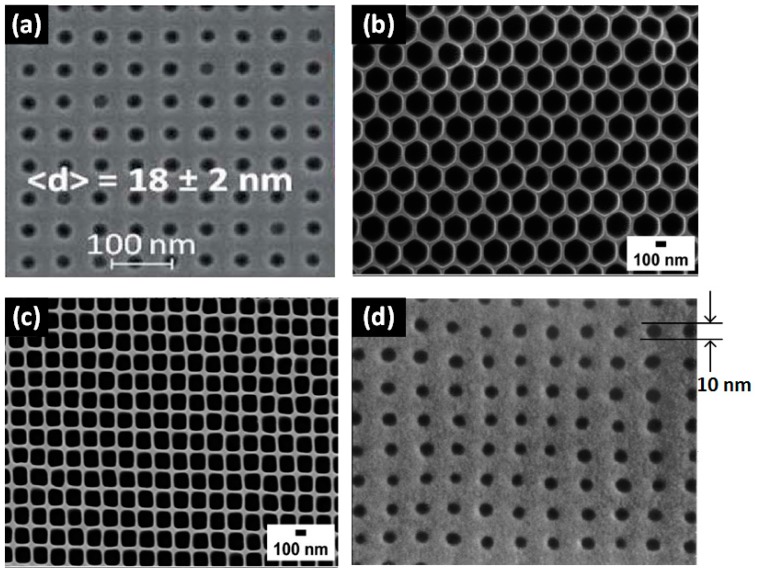
Nanopore arrays fabricated by electron beam lithography (EBL)-assisted fabrication technologies. (**a**) A nanopore array with an average diameter of 18 ± 2 nm, manufactured using the EBL-assisted RIE method (reprinted with permission from [86], copyright 2014, Royal Society of Chemistry). Ordered Al_2_O_3_ nanopores with (**b**) hexagonal and (**c**) square shapes prepared by nanoimprinting (reprinted with permission from [89], copyright 2010, American Chemical Society). (**d**) Nanopore arrays with a sub-10 nm diameter and 40 nm pitch fabricated by nanoimprinting (reprinted with permission from [88], copyright 1997, AIP Publishing).

Chemical etching techniques have the capability of fabricating high-density nanopores, but with pore sizes >20 nm, and the size uniformity needs to be improved. To address these limitations, electron beam lithography (EBL)-assisted etching technologies have been proposed. The EBL-assisted reactive ion etching (RIE) method was proposed by Han et al. [85] in 2006, where EBL was applied to form patterns on the membrane, followed by RIE to open nanopores. Using this method, Bai et al. [86] successfully manufactured nanopore arrays with uniform pore sizes (18 ± 2 nm) in a thin film embedded with metal electrodes, as shown in Figure 5a. Many groups fabricated nanopore arrays using nanoimprint technology with high-precision masks [87,88,89]. Notably, EBL should be applied to prepare high-precision masks to fabricate sub-100 nm nanopores. As shown in Figure 5b,c, ordered Al_2_O_3_ nanopores with square and hexagonal shapes were manufactured with the help of pre-patterned Al pillar structures using the nanoimprint method [89]. The RIE process was used to etch away the residual layer to form through-holes. Chou et al. [88] successfully fabricated sub-10 nanopores with the help of a chromium mask prepared by EBL (Figure 5d). Nanopore arrays obtained by EBL-assisted etching technologies possess uniform sizes and adjustable pore lengths. However, EBL requires a large up-front capital investment due to expensive instrumentation when compared with the standard photolithography. During the lithography process, EBL takes a long time (up to tens of hours) while standard photolithography takes only a few seconds to obtain wafer-scale patterns.

### 2.5. Metal Deposition and Heating Method

**Figure 6:**
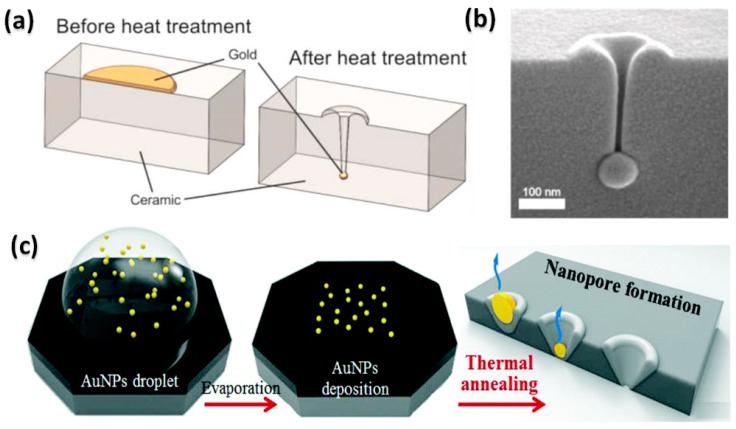
Metal deposition and the heating induced fabrication method. (**a**) Schematic and (**b**) SEM image of pore formation by heating gold patches on the ceramic membrane (reprinted with permission from [91], copyright 2015, American Chemical Society). (**c**) Schematic of nanopore formation via thermal annealing of gold nanoparticle (AuNP) droplets on the membrane (reprinted with permission from [92], copyright 2018, Royal Society of Chemistry).

Etching-based methods enable massive production of nanopores, as mentioned above. In fact, etching is not the only way to remove materials from substrates to fabricate nanopores. It was found by de Vreede et al. [90,91] that, when heated to close to their melting point, gold nanoparticles (AuNPs) moved perpendicularly into the substrate to generate nanopores, as shown in Figure 6a. In the manufacturing process, gold patches with a diameter of 1 µm and thickness of 18 nm were sputtered on the ceramic membrane by conventional photolithographic patterning. After the dewetting treatment, AuNPs formed and slowly moved into the substrate under heating. By controlling the temperature, nanopores of an extreme aspect ratio (diameter ≅ 25 nm, length up to 800 nm) were successfully produced [91] (Figure 6b). Park et al. [92] proposed a scalable fabrication of nanopores by thermal annealing AuNPs on a SiN membrane, as shown in Figure 6c. Instead of using photolithography, they deposited the AuNP-dispersed solution on the membrane for Au patterning. The distribution density of the attached AuNPs could be controlled by the pH value and concentration of the solution. After successive thermal annealing at 1060 °C, 8, 26, and 63 nm nanopores were formed by using the 50, 100, and 200 nm AuNPs. As opposed to the MaCE method, no chemical etchant is used in this method. 

According to Vreede [91] and Park [92], other metals can also be utilized to prepare nanopores using metal deposition and the heating method. Examples can be found in Au, Ni, Cu, and Mg on Al_2_O_3_ [93,94,95]. When heated to near the melting points of these metals, the Al_2_O_3_ membrane forms projecting edges and can thus create nanopores.

Heating-driven penetration of metal nanoparticles into membranes enables one-step fabrication of conical nanopores at wafer scales. However, it is difficult to control the pore size and distribution in an array with nanometer precision.

### 2.6. Methods to Shrink Nanopores

As discussed before, massively parallel fabrication technologies are not able to directly fabricate ordered <5 nm nanopore arrays. Thus, many advanced shrinking techniques have been proposed to contract the pores to desired sizes, such as FIB and FEB induced shrinkage [52,96,97,98,99,100,101,102], material deposition [103,104,105,106], and thermal oxidation [107,108]. Moreover, these shrinking technologies provide an opportunity to modify the surface properties because of the deposited material [103].

#### 2.6.1. FIB and FEB Induced Shrinkage Technology

As previously mentioned, FIB and FEB can be used for directly drilling nanopores on thin membranes. In fact, by adjusting the temperature, beam current, and spot, FIB and FEB can also shrink nanopores, namely FIB and FEB sculpting technology.

**Figure 7:**
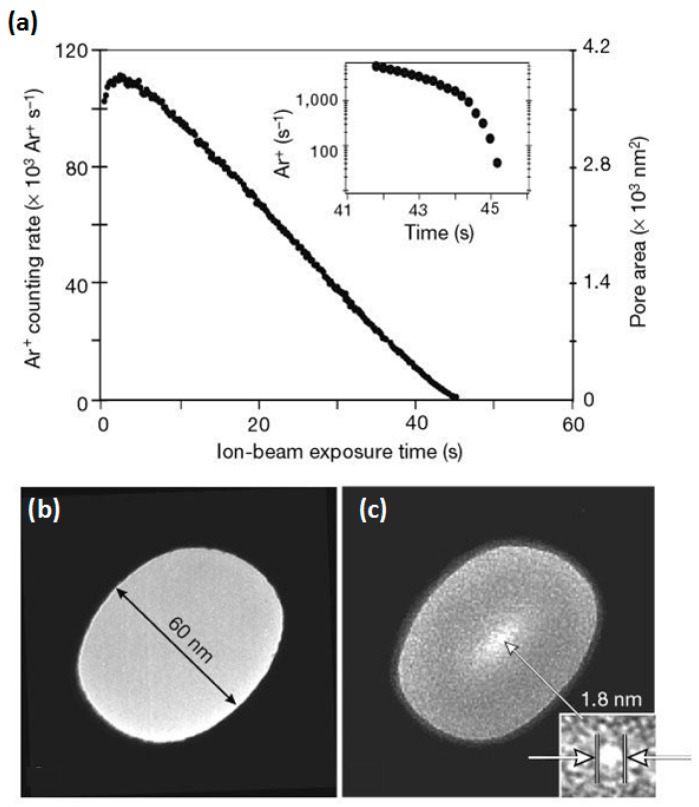
(**a**) Ar ion count rate and nanopore area vs. beam exposure time. Temperature, 28 °C. Flux, 28 Ar^+^/s/ nm^2^. Duty cycle, 200 ms/1 s. Energy, 3 keV. (**b**) TEM image of a nanopore (61 nm in diameter) before shrinking. (**c**) TEM image of the same sample after Ar^+^ exposure. The nanopore was shrunk to 1.8 nm (reprinted with permission from [52], copyright 2001, Springer Nature).

In 2001, the first solid-state nanopore was fabricated and shrunk to 1.8 nm by Li et al. [52] using the ion-beam sculpting method. They found that the nanopore diameter could either be reduced or enlarged by adjusting the velocity and temperature of the ion beam. According to their explanation, the flow and transport of the membrane material under the ion beam results in the shrinkage of the pore, while the high energy ion etching causes pore expansion. The nanopore size is affected by beam parameters, such as the energy, flux, and temperature [52]. Figure 7a shows the Ar^+^ count rate and nanopore area vs. beam exposure time. As a result, the pore size can be controlled precisely. As shown in Figure 7b,c, a nanopore with an initial diameter of 61 nm was shrunk to 1.8 nm.

**Figure 8:**
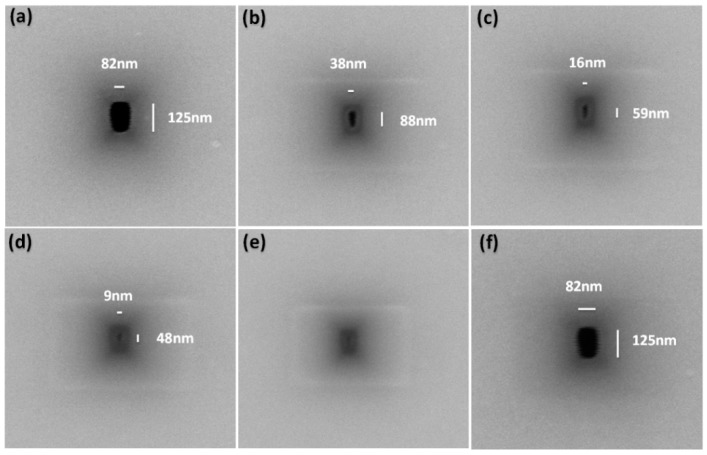
The shrinking process of a nanopore under electron beam irradiation from an SEM. The pore was irradiated for (**a**) 0 s, (**b**) 100 s, (**c**) 150 s, (**d**) 170 s, and (**e**) 200 s. (**f**) Nanopore recovery to its original size and shape after oxygen plasma cleaning (reprinted with permission from [102], copyright 2018, IEEE).

The FEB-induced shrinkage method utilizes a highly-accelerated electron beam via a TEM or a SEM with a visual feedback effect. In 2003, Dekker et al. [16] first used FEB sculpting to shrink nanopores. The relationship between the pore size and the beam parameters is similar to in FIB sculpting [16]. Chang et al. [101] used field emission SEM (FESEM) to successfully shrink nanopores on highly doped silicon-on-insulator (SOI) wafers. They deduced the mechanism to be the damage of Si/Si and Si/H bonds under electron beam irradiation. Other researchers believed that the nanopore shrinkage was caused by electron beam-induced deposition of materials [96,97,98,99,100,109]. Figure 8 shows a shrinking process of SEM-induced shrinkage of a nanopore [102]. The Si nanopore shrunk under the electron beam irradiation with time (Figure 8a–d), and closed (Figure 8e). After oxygen plasma cleaning, the nanopore recovered to its initial morphology and size, as shown in Figure 8f. According to the careful analysis by our group, the shrinkage mechanism is the electron beam induced deposition of hydrocarbon compounds in the SEM chamber. The pore shrinking rate is proportional to the electron beam current, and inversely proportional to the accelerating voltage and scanned area of the beam [98].

The FIB and FEB induced shrinkage technique can modify nanopores with sub-nanometer precision. However, the limited working space of FIB and FEB makes it difficult to increase the processing scale. Only one or several nanopores can be shrunk simultaneously under beam irradiation. 

#### 2.6.2. Material Deposition Induced Shrinkage Technology

**Figure 9:**
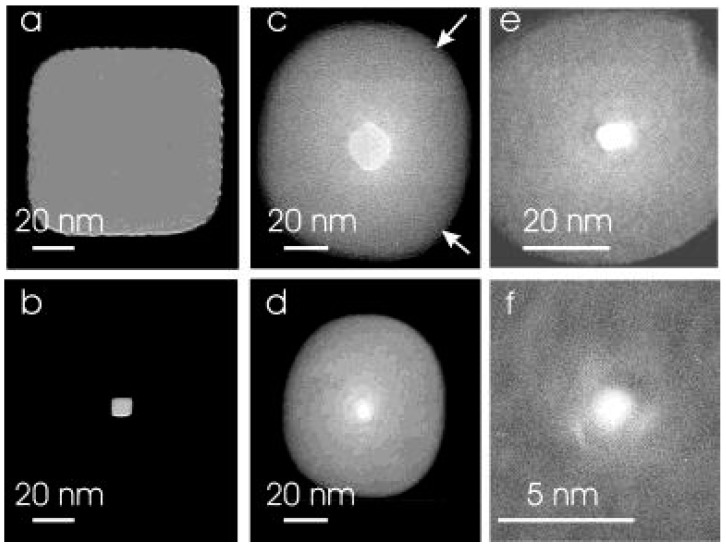
TEM images of nanopores before (top row) and after (below row) deposition of Al_2_O_3_ coatings by ALD. A square nanopore (**a**) before and (**b**) after 500 layers of Al_2_O_3_ coating. A circular pore with its initial diameter of 21.6 nm (**c**) before and (**d**) after 70 layers of Al_2_O_3_ coating to produce a ~4.8 nm pore. A nanopore with its initial diameter of ~7 nm (**e**) before and (**f**) after 24 layers of Al_2_O_3_ coating to produce a ~2 nm pore (reprinted with permission from [103], copyright 2004, American Chemical Society).

Depositing materials on the inner-surfaces of nanopores can contract numerous nanopores simultaneously. Techniques, such as ALD [103], evaporation [104], electroplating [105], and plasma-enhanced chemical vapor deposition (PECVD) [106], have been used to shrink nanopores. Atomic resolution and high conformity could be realized using the ALD technique. Materials that can be deposited by ALD include Al_2_O_3_, HfO_2_, and TiO_2_. Al_2_O_3_ nanopores have positive surface charge densities when immersed into buffer solutions for DNA detection. Thus, they have been used to slow down the DNA translocation speed based on the electrostatic interactions between the pore surfaces and the negatively charged DNA molecules [103]. HfO_2_ and TiO_2_ are high dielectric-constant materials, which exhibit excellent electrical and mechanical properties and have deposition temperatures as low as 150 °C [9]. Chen et al. [103] successfully shrank SiN nanopores with diameters of 70 to 100 nm to any desired size by depositing various layers of Al_2_O_3_, as shown in Figure 9. They found that the 1/f noise (pA^2^/Hz) of the ALD-Al_2_O_3_ coated nanopore was reduced by nearly four orders of magnitude compared with the SiN nanopore without Al_2_O_3_ coatings [103].

The material deposition method is an effective and parallel shrinkage technique. The deposited material can modify the nanopore surface charge density, reduce the noise, and slow down the translocation speed of DNA in nanopore-based detection [103]. However, the deposited material increases the membrane thickness, inducing an increase of the effective length of the nanopore.

#### 2.6.3. Thermal Oxidation Induced Shrinkage Technology

**Figure 10:**
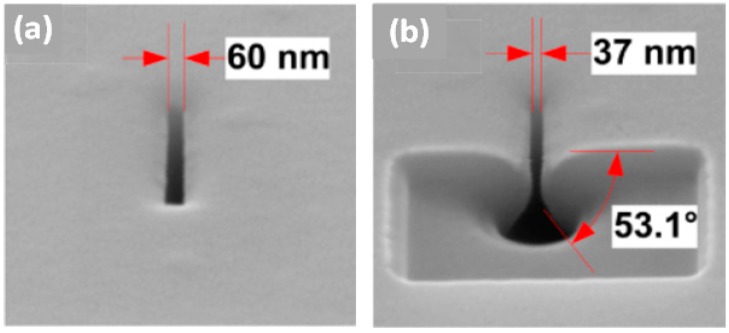
SEM micrographs of a nanopore (**a**) before and (**b**) after thermal oxidation-induced shrinkage. FIB cutting was conducted to view the cross-sectional morphology of the shrunk pyramidal nanopore (reprinted with permission from [108], copyright 2013, IOP Publishing).

Thermal oxidation is a simple and effective shrinking method for Si nanopores only. In 2011, Asghar et al. [107] proposed a method to directly heat and shrink SiO_2_ nanopores. When the temperature is higher than 1000 °C, the SiO_2_ film softens, and atoms subsequently diffuse to a region where the surface energy is lower, resulting in the pore shrinkage. Deng et al. [108] fabricated pyramidal Si nanopores by the wet etching method, and then shrunk the pores using dry oxygen oxidation (Figure 10). Nevertheless, nanopores were shrunk at a very low average shrinkage rate (4.6 nm/h), and the viscous flow of SiO_2_ changed the cross-sectional morphology of the nanopore. As shown in Figure 10b, the pyramidal tip became rounded compared to the sharp included angle of 54.74° obtained from anisotropic etching of Si.

To summarize, all massive fabrication technologies are not able to directly fabricate ordered <5 nm nanopore arrays, since they lack nanometer-precision. The EBL-assisted method can fabricate nanopore arrays with a pore size of 10 nm, but it requires costly EBL technology. Shrinking technologies can not only reduce the pore size, but also modify nanopore properties, such as the surface charge property, 1/f noise, and the influence on the DNA translocation, because of the deposited materials. FIB and FEB induced shrinkage, material deposition, and thermal oxidation technologies can contract numerous nanopores at a time. Besides, the available minimum pore size, the inner morphology of the pore, and the fabrication scale vary among different technologies, as summarized in Table 1. 

Using these fabrication techniques, nanopores with various sizes and morphologies can be prepared and used for different applications. Compared with cylindrical nanopores, conical nanopores have ion-selective transport characteristics, which hold great potential in the applications of seawater desalination, energy conversion, and ion logic circuits. Details about the applications of conical nanopores are provided in Section 3.3.

## 3. Applications

### 3.1. DNA Sequencing

Nanopore-based DNA sequencing has been popular among researchers due to its cost-effectiveness, label-free process, long read length, and no need for amplification [21]. DNA homopolymer discrimination using the blocked ionic current signals of DNA homopolymers (poly(A), poly(T), etc.) via solid-state nanopores has been realized [117,118,119]. However, DNA sequencing at single-nucleotide precision has not been achieved so far based on solid-state nanopores, as many challenges exist in both experimental and theoretical studies, such as the limited spatial and temporal resolution [17,120,121,122], pore clogging [123,124], and stochastic DNA motion [125,126]. More details about the advances and challenges of solid-state nanopores for DNA sequencing can be found in previous reviews [2,120,127,128,129,130]. Herein, we mainly discuss the spatial and temporal resolutions of DNA sequencing via solid-state nanopores.

**Figure 11:**
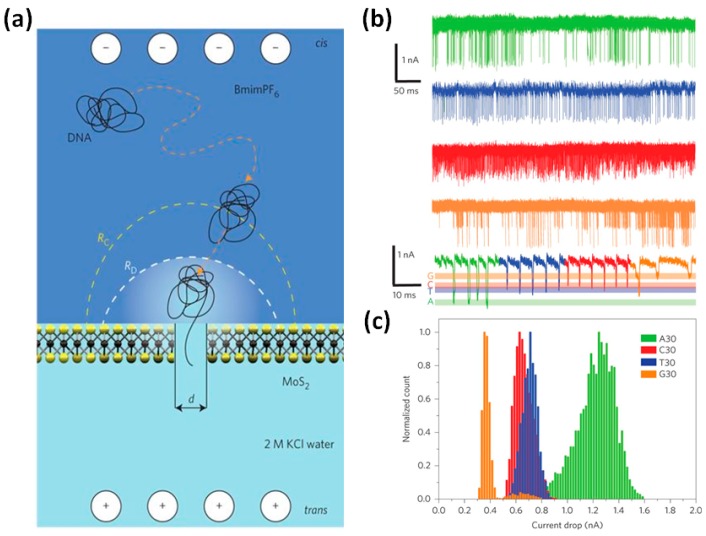
Identification of single nucleotides in MoS_2_ nanopores. (**a**) Schematic of the DNA homopolymer translocation detection. The cis reservoir contains room temperature ionic liquids (BmimPF6) and the trans reservoir contains 2 M aqueous KCl solution. Two reservoirs are separated by a monolayer MoS_2_ membrane with a nanopore. (**b**) Translocation signals for each DNA homopolymer in a 2.8 nm MoS_2_ nanopore: poly A30 (green), poly C30 (red), poly T30 (blue), and poly G30 (orange). (**c**) Normalized histogram of current drops for each DNA homopolymer (reprinted with permission from [119], copyright 2015, Springer Nature).

The use of nanopores fabricated in ultra-thin 2D membranes is a strategy for improving the spatial resolution. For instance, the thickness of a single-layer graphene is 0.335 nm, which is comparable to the height of a nucleotide (0.34 nm) in a DNA strand [56]. The first studies on graphene nanopores were published in 2001 [112,131]. Later, other 2D materials, such as boron nitride (BN) [113,114] and molybdenum disulfide (MoS_2_) [111,119], have been studied. However, the interactions between DNA and graphene surfaces may cause severe nonspecific adsorption of DNA and pore clogging [128]. It is reported that the interaction between DNA and the pore surface has been successfully decreased using MoS_2_ nanopores [132]. Feng et al. [119] performed DNA homopolymer translocations with a 2.8 nm MoS_2_ nanopore. DNA homopolymer discrimination was achieved successfully by the detection of free nucleotides, as shown in Figure 11. High viscosity room temperature ionic liquids were used to control the speed of nucleotide translocation.

Temporal resolution is another big challenge for solid-state nanopore-based DNA sequencing. The appropriate DNA translocation speed is 1 bp/ms to achieve single nucleotide resolution [3]. Biological nanopores have already reached velocities below this value [3], while the speed of DNA translocation through solid-state nanopores is between two to four orders of magnitude faster than the ideal velocity [17,121,122,133,134]. Various methods have been developed to slow down the translocation, such as DNA modification, nanopore functionalization, and control of the applied electric field, solution parameters, and external pressure, as summarized in Table 2. 

### 3.2. Single Molecule Detection

**Figure 12:**
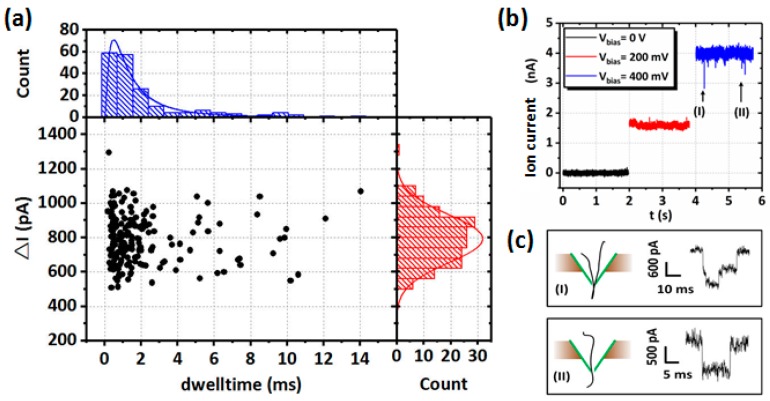
Experimental results of λ-DNA translocation through a 5.8 nm nanopore on the pyramidal membrane. (**a**) Scatter plot of ionic current blockade (ΔI) vs. dwell time (Δt) of double-strand DNA (dsDNA) translocation events. The top figure presents the histogram of Δt, and the right figure shows the histogram of ΔI. (**b**) Time traces of λ-DNA translocation events through the pore under different applied voltages (V_bias_). (**c**) Schematic and time traces of λ-DNA translocation events in (I) folded and (II) linear modes (reprinted with permission from [63], copyright 2018, American Chemical Society).

**Figure 13:**
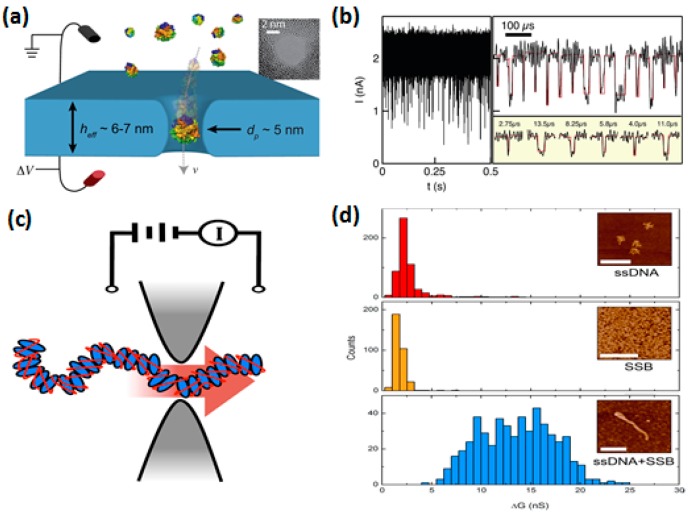
Protein detection using solid-state nanopores. (**a**) Schematic of the protein transport through a 5-nm-diameter HfO_2_ nanopore. The inset shows the TEM image of the HfO_2_ pore. (**b**) Current vs. time trace of the protein at the applied voltage of –125 mV (reprinted with permission from [154], copyright 2014, Elsevier). (**c**) Schematic of the detection of a single-stranded binding protein (SSB) to ssDNA using a SiN nanopore. (**d**) Histograms of the mean amplitude of electrical conductance (ΔG) for ssDNA alone (red), SSB alone (yellow), and the mixture of ssDNA with SSB (blue). Insets demonstrate the atomic force microscope images of the respective molecules. Scale bars represent 400 nm (reprinted with permission from [158], copyright 2015, American Chemical Society).

As promising sensors for single-molecule analysis, nanopores provide a highly confined space within which the single molecule characteristics can be efficiently converted into the measurable electrochemical signal. The principle of solid-state nanopore-based single molecule detection is to measure the blocked ionic current (ΔI) and dwell time (Δt) when molecules are passing through a nanopore [23,24,25]. Our group demonstrated λ-DNA translocation experiments through nanopores on pyramidal membranes [63]. Figure 12a shows the scatter plot of ΔI versus Δt. The amplitude of ΔI increased with the applied voltages (V_bias_), as shown in Figure 12b. According to the analysis of the time traces, λ-DNA translocation in folded and linear modes were distinguished (Figure 12c). Apart from DNA translocation, a large number of studies have been focused on DNA methylation detection [150,151], and interactions between DNA and pore surfaces [128,152]. Solid-state nanopores with larger pore sizes have also been used to detect larger molecules, such as proteins [85,153,154] and protein–DNA complexes [155,156,157,158,159]. Voltage-driven translocation of proteins through nanopores in ultrathin HfO_2_ and SiN membranes was conducted by Larkin et al. [154], where sub-30 kDa proteins and their complexes were efficiently detected, as shown in Figure 13a,b. They conducted volumetric measurements of the two proteins using the Coulter theory, and found that the measured protein diameters coincide well with hydrodynamic diameters obtained from dynamic light-scattering measurements. Using a 40 nm SiN naonopore, Plesa et al. [160] successfully detected five kinds of proteins: aprotinin (6.5 kDa), ovalbumin (6.5 kDa), beta-amylase (45 kDa), ferritin (200 kDa), and thyroglobulin (660 kDa). Besides proteins, DNA–protein interactions have been studied as well with solid-state nanopores. Marshall et al. [158] studied the binding of a protein to DNA using a solid-state nanopore, as shown in Figure 13c. The binding event was confirmed by a shift in the mean amplitude of the electrical conductance (ΔG) (Figure 13d). Apart from biomolecules, solid-state nanopores have been utilized as platforms for the detection of other particles, such as microgels [161] and polystyrene nanoparticles [162].

Single molecule detection via solid-state nanopores has made great progress. The passing modes of DNA molecules (linear, partially folded, and completely doubled over molecules) have been successfully detected [17,23,25,43,163,164]. Additionally, the characterization and detection of molecules has been realized based on the diverse properties of the detected molecules, such as the net charge [165,166], molecular weight [167], volume [154], and binding events [158]. These developments have been directed toward more sensitive detection of molecules with smaller feature sizes or complex structures.

### 3.3. Ion-Selective Transport

Inspired by biological membranes, various “smart” solid-state nanopores have been proposed to imitate and investigate the transmembrane transport of specified ions. In a nanofluidic system, the ion-selective transport is caused by the asymmetric transport of ions through a nanopore due to the electric double layer (EDL) [168,169]. Based on this specific property, solid-state nanopores have been used for applications in water desalination [170,171], ion logic circuits [172,173], energy conversion [37,174], and molecular separation [175].

**Figure 14:**
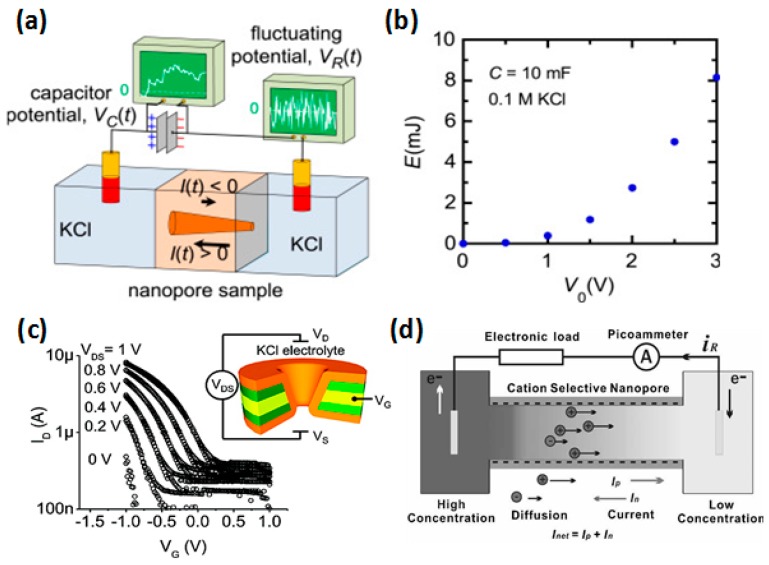
Applications based on ion-selective transport properties of the solid-state nanopores. (**a**) Schematic of the experimental set-up for energy conversion from fluctuating signals using a conical nanopore. (**b**) Energy (E) stored in the load capacitor as a function of the noise voltage amplitude (V_0_) in the 0.1 M KCl solution at C = 1 mF (reprinted with permission from [174], copyright 2015, Elsevier). (**c**) Log plot of ionic current (I_D_) vs. the gate voltage (V_G_) of a nanopore. Inset demonstrates the schematic of the electrode-embedded nanopore (reprinted with permission from [173], copyright 2009, American Chemical Society). (**d**) Schematic of the generation of net current (I_net_) in a solution with a concentration gradient (reprinted with permission from [178], copyright 2010, WILEY-VCH Verlag GmbH & Co. KGaA, Weinheim).

The ion-selectivity of the nanopore is influenced by the size, geometry, and wall surface charges of the pore. Energy conversion from external fluctuating signals (white noise) through an asymmetric nanopore was studied theoretically and experimentally [174]. Figure 14a shows the schematic of the experimental set-up for capacitor charging using a conical nanopore immersed in KCl solution. Figure 14b shows the energy stored in the load capacitor as a function of the noise voltage amplitude (V_0_) in the 0.1 M KCl solution. Gate modulation of the nanopore wall surface charges affects the ion transport behavior, forming the voltage-gated selective ion channel. Li et al. [176] discussed electric field-controlled ion transport in a TiO_2_ nanochannel. Ionic field-effect transistors with sub-10 nm nanopores were investigated via conical nanopores [173], as shown in Figure 14c. Electrical control of the DNA translocation speed was realized by gate modulation of a nanopore because of the surface charge induced dragging forces on DNA [177]. Solution conditions affect ion-selective transport through a nanopore as well. By applying a concentration gradient, nanopores are able to convert salinity gradient energy to electric energy [178,179], as shown in Figure 14d. The pH value of the solution is able to regulate the ion transport, for it influences the surface charge property of the nanopore [179,180].

Conical solid-state nanopores have abilities to actively manipulate and control the transport of ions and molecules. They can be utilized to mimic the transmembrane motion of ions and molecules in protein or biological channels, while providing superior mechanical, thermal, and chemical stabilities than the biofilms. In the future, more modifications of nanopores should be done to increase the sensitivity. Meanwhile, scalable fabrication of nanopores with various shapes and sizes can improve the efficiency of energy storage and conversion.

### 3.4. Nanopatterning

**Figure 15:**
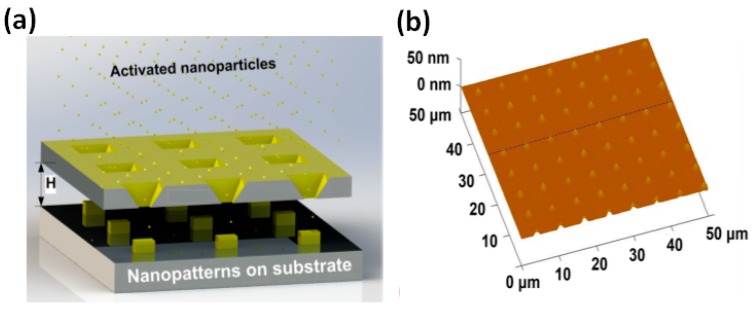
Nanopatterning with solid-state nanopores. (**a**) Schematic of nanopatterning by the deposition of activated gold-palladium (Au-Pd) nanoparticles on the substrate with a Si nanopore array. (**b**) An atomic force microscope (AFM) image of a deposited Au−Pd nanocube array with an average planar size of 300 nm (reprinted with permission from [184], copyright 2014, American Chemical Society).

Surface nanopatterning has become one of the most popular research topics in the fields of microelectronics, materials science, and engineering. EBL is widely used because of its high precision and flexibility to create patterns and structures with various shapes and dimensions [85]. However, structures are patterned one by one, making it costly for massive preparation. Besides, EBL needs to complete pattern preparation on conductive substrates. Nanopatterning with nanopores as reusable masks is a kind of shadow-mask patterning technique. It does not require any photoresist, so it has the advantage of simplifying the fabrication process and shaping the nanostructure array on any substrate. Nanopores in Al_2_O_3_ and Si membranes have been widely used in the fabrication of quantum dots [181], nanotubes [182,183], nanocubes [184], and nanoplasma devices [185]. Wu et al. [181] fabricated ultrathin alumina membranes (UTAMs) with a thickness of about 80 nm by a two-step anodization process. They utilized the UTAMs as masks to pattern ordered arrays of Au nanodots with diameters of about 20 nm. Figure 15a shows the schematic illustration of nanopatterning with Si nanopores as masks by our group [184]. By direct deposition of gold-palladium (Au-Pd) nanoparticles, nanocube arrays with an average size of 300 nm were fabricated on the Si substrate (Figure 15b). The thickness of the Si membrane containing the nanopore arrays was ~3 µm, which can provide more robustness than the 80 nm-thick UTAMs. Moreover, solid-state nanopore arrays can be prepared by scalable fabrication technologies with low cost. The challenge lies in the preparation of highly uniform nanopore arrays with nanometer precision.

## 4. Challenges and Future Outlook

There are challenges in the fabrication of nanopores and the accuracy and sensitivity of nanopore-based detection. Each fabrication method has its advantages and disadvantages. With the demand of massively parallel sensing and detection of smaller molecules, a rapid and cost-effective scalable technology for the manufacturing of ordered nanopore arrays with small sizes (<10 nm) and better uniformity should be developed and optimized.

With respect to the accuracy and sensitivity of solid-state nanopore-based detection, many challenges exist in both experimental and theoretical studies, such as limitations in the spatial and temporal resolutions [17,120,121,122], pore clogging [123,124], and stochastic DNA motion [125,126]. Great efforts have been made to solve these problems, including shorting the effective pore length [56,114], slowing down the DNA translocation speed [133,138,139,141], modifying nanopore surfaces [79,110], and controlling the DNA motion [135,137]. Nanopore functionalization is a useful strategy to increase the sensitivity and diversity of nanopore-based detection. Covalent nanopore modification strategies include the immobilization of antibodies [186], decoration of DNA hairpins [140], and the use of a nanofiber mesh [136] on the nanopore surfaces. Non-covalent nanopore modification via self-assembly of pyrene ethylene glycol (PEG) was demonstrated to prevent DNA from adsorbing on graphene [123]. More functionalization strategies of solid-state nanopores can be found in many other reviews [40,41,128].

**Figure 16:**
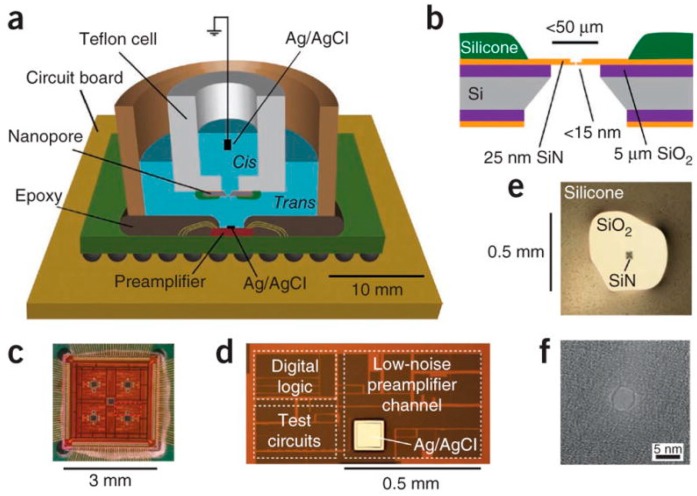
(**a**) S Schematic of a CMOS-integrated nanopore platform (CNP). (**b**) Cross-section schematic of the nanopore. (**c**) Optical micrograph of the 8-channel CMOS current preamplifier. (**d**) One channel of the preamplifier. (**e**) Optical image of a SiN nanopore. (**f**) A TEM image of a SiN nanopore with a diameter of 4 nm (reprinted with permission from [187], copyright 2012, American Chemical Society).

Though various issues still need to be completely resolved, solid-state nanopores still hold potential for DNA sequencing and other applications with the development of nanotechnology and biotechnology. Integration nanopores with readout integrated circuits (ICs) can meet future requirements for miniaturization, lightweight properties, and low power consumption. Jacob et al. [187] fabricated a complementary metal-oxide-semiconductor (CMOS)-integrated nanopore platform (CNP) with a bandwidth >1 MHz and an SNR > 5, as shown in Figure 16. A sub-microsecond temporal resolution for DNA sequencing was achieved. Additionally, the measured high frequency noise of the CNP (3.2 pA rms at 100 KHz bandwidth, 24 pA rms at 1 MHz bandwidth) is smaller than the Axon 200B patch clamp amplifier (9 pA rms at 100 KHz bandwidth and 247 pA rms at 1 MHz bandwidth). Recently, Shekar et al. [188] successfully increased the CNP bandwidth to an unprecedented 10 MHz in the low noise recording mode, and they were able to achieve an SNR > 10 at a measurement bandwidth of 5 MHz during ssDNA translocations. Nanopores integrated with CMOS amplifiers have improved both the SNR and the temporal resolution.

## 5. Conclusions

This paper summarizes advances in the fabrication and shrinking technologies as well as applications of solid-state nanopores. Solid-state nanopores perform as versatile platforms thanks to their superior performances, such as modified pore sizes, morphologies, and surface charge properties, when compared with biological nanopores. As a fundamental issue, the fabrication of solid-state nanopores has gained extensive attention among researchers. FIB and FEB drilling technologies hold possibilities for the fabrication of nanopores with atomic resolutions in a serial manufacturing way. Dielectric breakdown emerges as a novel method to prepare nanopore in thin membranes with low cost, but also lacks the capability of massive production. Chemical solution etching methods, EBL-assisted methods, metal deposition, and heating methods have been proposed for the requirement of scalable fabrication. However, the available minimum pore size, size uniformity in the array, and the manufacturing cost should be balanced. Abundant shrinking strategies have been introduced to modify the size, morphology, and surface properties of the nanopore. These shrinking strategies can assist in the fabrication of solid-state nanopores with desired sizes and shapes. After a detailed discussion and comparison of the fabrication technologies reported to date, we reviewed the applications of solid-state nanopores in DNA sequencing, single molecule detection, ion-selective transport, and nanopatterning. In our prospects, though there are still many challenges, solid-state nanopores will continue to be an active and engaging research area.

## Figures and Tables

**Table 1:** Summary of the fabrication methods for solid-state nanopores.

Method	Material	Minimum Diameter	Inner Morphology	Fabrication Scale	Controllability/Reproducibility	Manufacturing Cost	Manufacturing Time/Rate
Focused ion/electron beam (FIB/FEB) drilling	SiN [16,42,43,44,45,54], SiO_2_ [46], Metallic and metal oxide [24,47,110,111], 2D materials [56,112,113,114]	1.3 nm [54]	Cylindrical/Hourglass	One at a time	Good controllability (nanometer precision)	High (requires FIB or TEM)	0.2 s for one pore [45]; hours for a high-density array
Dielectric breakdown	SiN [59,60], SiO_2_ [63], HfO_2_ [64]	<1 nm [60]	Cylindrical	One/multiple at a time	Poor controllability over the amount of nanopores	Low (requires a voltage source and electrolyte solutions)	~ 1 h for one or multiple pores [60]
Metal-assisted chemical etching (MaCE)	Si [74,76,77,84]	50 nm [84]	Conical	Array (pore density of 10^9^ cm^−2^) [84]	Poor distribution in the pore size and location	Middle (requires heavy metal particles, HF/H_2_O_2_ solutions)	30 min to etch 30 µm-thick Si, and 24 h to etch 500 µm-thick Si [84]
Electrochemical anodization	Metal oxide [70,71,72,73]	23 nm [73]	Cylindrical/hexagonal prism	Array (pore density of 5 × 10^10^ cm^−2^) [73]	Good controllability when fabricating >20 nanopores	Low (requires a voltage source and etchant solutions)	Etching rate of 40 μm/h at 30 °C under bias voltage of 70 V [73]
Ion-track etching	Polymers [67,68,69,82,83,115]	51 nm (in an array) [82], 2 nm (individual pores) [83]	Cylindrical	Array (pore density of 10^7^ to 10^9^ cm^−2^) [82]	Poor distribution in the pore size (standard deviation of the pore size was 22% and 25% [82])	Middle (requires heavy ion accelerometers)	UV radiation for 10 to 24 h, and NaOH etching for 5 min on the 23 µm-thick PET [82]
Feedbackcontrolled wet etching	Si [65,66]	30 nm [66]	Truncated-pyramidal	Array (pore density of 1.96 × 10^6^ cm^−2^) [66,78]	Poor size uniformity for ~30 nm nanopores [66]. Good size uniformity (± 5%) for >500 nm nanopores [78].	Low (requires heated KOH etchant)	84 µm/h in 33 wt % KOH at 80 °C
EBL-assisted RIE	SiN [85], SiO_2_ [86,116]	18 nm [86]	Cylindrical	Array (pore density of 5 × 10^10^ cm^−2^) [86]	Good size uniformity of 18 ± 2 nm.	High (requires EBL technique)	Hours to form patterns at the wafer-scale via EBL
EBL-assisted nanoimprint	Polymer [88], Al_2_O_3_ [89]	10 nm [88]	Cylindrical/hexagonal prism	Array (pore density of 2.6 × 10^11^ cm^−2^) [88]	Good controllability thanks to the high-precision EBL	High (EBL is more expensive than the photolithography technique)	Hours to form high-precision masks at the wafer-scale via EBL
Metal deposition and heating	SiN [91,92], SiO_2_ [91]	8 nm [90]	Conical	Array (4 × 10^6^ cm^−2^) [90]	Poor distribution in the pore size and location	Middle (requires metal nanoparticles and a furnace)	Several hours for heating Au at 1067 ± 5 °C [90]
Shrinking by FIB/FEB	SiN [52,96], Metal oxide [97], Si [98,99], SiO_2_ [100]	<1 nm	Cylindrical/conical	One or several pores at a time	Sub-nanometer precision	High (requires FIB, SEM or TEM)	Shrinking rate of 0.67 nm/s [98]
Shrinking by material deposition	Al_2_O_3_ [103], SiN [106]	<1 nm	Cylindrical/conical	Wafer scale	Good (ALD has sub-nanometer precision [103])	Middle (depends on the deposition technology)	Shrinking rate of 0.1 nm/cycle by ALD (1 cycle takes several seconds)
Shrinking by thermal oxidation	Si [107,108]	<1 nm	Cylindrical/conical	Wafer scale	Good	Middle (requires an oxidation furnace)	Shrinking rate of 4.6 nm/h [108]

**Table 2:** Methods to slow down the DNA translocation speed.

Modified Object	Methods	Maximum Scaling Down
DNA molecules	(1) Insert macromolecules into the DNA sequence. (2) Add a protein to the end of a DNA sequence to specifically bind to molecules on the pore. (3) Control DNA motions with optical [135,136] or magnetic [137] tweezers.	Five orders of magnitude [135]
Solid-state nanopores	(1) Reduce the pore size to improve contact friction [138,139]. (2) Gate modulation [140]. (3) Coating a neutral HfO_2_ membrane [24], a positively charged Al_2_O_3_ membrane [103,110], a nanofiber mesh [141], or a PH-sensitive polymeric cushion [131]. (4) Decorate with DNA hairpins or aptamers [140].	Two orders of magnitude [141]
Applied electric field	Modulate the electric field polarity [32].	In simulation
Electrolyte solution	(1) Increase solution viscosity [142]. (2) Control the temperature [143] and salt concentration [144]. (3) Change the solute, where the translocation speed is represented by KCl > NaCl > LiCl [145,146].	One order of magnitude [142,145]
External pressure	Apply a stress opposite to the electric field force [147,148,149].	One order of magnitude [147]
